# Characterization of cervical fluid *Ureaplasma* species in pregnant women with spontaneous preterm delivery

**DOI:** 10.1038/s41598-025-16612-2

**Published:** 2025-08-30

**Authors:** Radka Bolehovska, Antonin Libra, Rudolf Kukla, Pavel Bostik, Ivana Musilova, Jana Matulova, Mariusz Grzesiak, Bo Jacobsson, Marian Kacerovsky

**Affiliations:** 1 Institute of Clinical Microbiology, University Hospital, Hradec Kralove, Czech Republic; 2 Institute of Clinical Microbiology, Faculty of Medicine in Hradec Kralove, Charles University, Hradec Kralove, Czech Republic; 3 Biomedical Research Center, University Hospital Hradec Kralove, Hradec Kralove, Czech Republic; 4 Generi Biotech, Hradec Kralove, Czech Republic; 5 Department of Obstetrics and Gynecology, University Hospital Olomouc, Zdravotniku 248/7, Olomouc, Czech Republic; 6 Department of Obstetrics and Gynecology, Hospital Most, Krajska zdravotni a.s., Most, Czech Republic; 7 Department of Non-Medical Studies, Faculty of Medicine in Hradec Kralove, Charles University, Hradec Kralove, Czech Republic; 8 Department of Perinatology, Obstetrics and Gynecology, Polish Mother’s Memorial Hospital, Lodz, Poland; 9 Department of Gynecology and Obsetrics, Medical University of Lodz, Lodz, Poland; 10 Department of Perinatology, Obstetrics and Gynecology, Institute of Clinical Science, Sahlgrenska Academy, University of Gothenburg, Gothenburg, Sweden; 11 Department of Obstetrics and Gynecology, Region Västra Götaland, Sahlgrenska University Hospital, Gothenburg, Sweden; 12 Department of Genetics and Bioinformatics, Domain of Health Data and Digitalisation, Institute of Public Health, Oslo, Norway

**Keywords:** Amniotic fluid, Invasive sampling, Genital mycoplasmas, Non-invasive sampling, Preterm birth, Clinical microbiology, Outcomes research

## Abstract

*Ureaplasma* spp., commonly identified in the vagina/cervix of pregnant women with spontaneous preterm delivery, are the most frequently detected microorganisms in amniotic fluid. To date, 14 *U.* spp. serotypes have been characterized; however, modern molecular biology methods can distinguish different *U.* spp genotypes. Considering these factors, a knowledge gap exists regarding the association between *U.* spp. genotypes and the risk of the ascension of *U.* spp. from the cervix to the amniotic cavity. To fill this gap, an expanded multilocus sequence-typing scheme of *U.* spp. was performed to assess the relationship between cervical and amniotic fluid *U.* spp. in pregnant women with spontaneous preterm delivery. This study included 109 and 69 pregnant women with spontaneous preterm labor (PTL) and preterm prelabor rupture of membranes (PPROM), respectively. *U.* spp. DNA in cervical fluid was identified in 49% and 55% of the women with PTL and PPROM, respectively. The concurrent presence of *U.* spp. DNA in amniotic fluid was observed in 17% and 59% of the pregnant women with PTL and PPROM, respectively. Among pregnant women with PTL and PPROM, 38 expanded sequence types of cervical *U*. spp. were identified. No associations were observed between specific genotypes, subgroups, or clusters of cervical *U.* spp. and the presence of amniotic fluid *U*. spp. in pregnant women with spontaneous preterm delivery.

**Supplementary Information:**

The online version contains supplementary material available at 10.1038/s41598-025-16612-2.

## Introduction

Spontaneous preterm delivery is responsible for approximately two-thirds of all preterm deliveries (delivery prior to the 37th week of gestation)^1^. This subset of preterm deliveries consists of two main phenotypes with different clinical presentations: (i) spontaneous preterm labor (PTL) with intact membranes (regular uterine activity accompanied by changes in the cervix) and (ii) preterm prelabor rupture of membranes (PPROM) (rupture of fetal membranes with leakage of amniotic fluid before the onset of regular uterine activity)^1^.

Regardless of the substantial differences in their underlying pathophysiologies^2,3^, both spontaneous preterm delivery phenotypes are often complicated by the presence of microorganisms and/or elevated levels of inflammatory mediators in the amniotic fluid^4–7^. The most common microorganisms implicated in these complications are *Ureaplasma* spp.^8–12^.

*U.* spp. include small, low-virulence microorganisms without bacterial cell walls, with 14 different currently known serovars^13,14^. Nevertheless, modern diagnostic approaches can dissect more *U.* spp. genotypes^15–17^. For example, an expanded multilocus sequence-typing (eMLST) scheme can identify dozens of expanded sequence types (eST) of *U.* spp. based on nucleotide differences between four housekeeping genes and two putative virulence genes^16^. Recently, 33 eSTs of *U.* spp. isolated from the amniotic fluid of pregnant women with PPROM have been characterized^18^.

*U.* spp. commonly colonizes the cervix and/or vagina of pregnant women with spontaneous preterm delivery^11,12,19,20^. This colonization is more frequent in pregnant women with spontaneous preterm delivery than in those who deliver at term^11,14,20,21^. Colonization of the cervix and/or vagina by *U*. spp. is related to (i) a higher prevalence of microorganisms and/or their nucleic acids in the amniotic fluid in both PTL and PPROM^11,12^; (ii) a higher rate of sterile intra-amniotic inflammation (elevated levels of inflammatory mediators in the amniotic fluid without concomitant presence of microorganisms and/or their nucleic acids) in pregnant women with PTL^12^; and (iii) a higher frequency of acute inflammatory lesions of the placenta in PPROM^11^.

Considering the various serotypes/genotypes of *U.* spp.^13,15^ and the fact that intra-amniotic complications caused by *U.* spp. develop only in a subset of pregnant women with cervical/vaginal colonization of *U.* spp.^11,12,22^, the question of the pathogenicity of *U.* spp. in relation to PTL or PPROM remains uresolved, as no causal association has yet been demonstrated. Current evidence regarding the association between the vaginal presence of specific serotypes of *U*. spp. and the subsequent risk of spontaneous preterm delivery is conflicting^19,21^.

To fill this knowledge gap, the eMLST scheme was performed on the cervical and amniotic fluids *U.* spp. DNA obtained from pregnant women with both phenotypes of spontaneous preterm delivery with the following aims: (i) to characterize eSTs of *U.* spp. isolated from the cervical fluid of pregnant women with PTL and PPROM, (ii) to evaluate differences in maternal demographic and clinical parameters between clusters and phylogenetic subgroups of cervical fluid *U.* spp. from pregnant women with PTL and PPROM, and (iii) to compare the eSTs of *U.* spp. DNA between paired amniotic and cervical fluids *U.* spp. in pregnant women with PTL and PPROM.

## Results

**Fig. 1:**
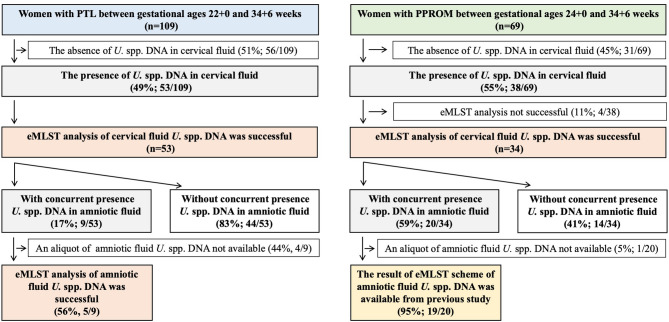
A flowchart illustrating the selection process of pregnant women with preterm labor with intact membranes and preterm prelabor rupture of membranes included in the study.

The study included 109 and 69 pregnant women with PTL and PPROM, respectively. Figure 1 shows a flowchart of the pregnant women enrolled in this study. No difference was observed regarding the presence of *U.* spp. DNA in the cervical fluid between the pregnant women with PTL and PPROM (49% [53/109] vs. 55% [38/69];* p* = 0.44; see Fig. 1). Demographic and clinical characteristics of the pregnant women with PTL and PPROM regarding the presence and absence of cervical fluid* U*. spp. DNA are shown in Supplementary Tables 1 and 2.

Exact characterization of all six loci of the eMLST scheme was successful in 100% (53/53) and 90% (34/38) of the aliquots of the cervical fluid *U.* spp. DNA extracted from pregnant women with PTL and PPROM, respectively; see Fig. 1. Regarding the aliquots of cervical fluid *U.* spp. DNA from pregnant women with PTL and PPROM, 38 different eSTs were identified (Supplementary Table 3), 11 of which were novel (#49, 245–247, 252, 254, 256–258, 260, and 261) and were added to the PubMLST database. The most frequent eSTs in the cervical fluid *U.* spp. DNA were #41, #20, and #244, found in 10% (n = 9), 8% (n = 7), and 8% (n = 7) of the aliquots, respectively.

Pregnant women with PTL and confirmed cervical fluid *U*. spp. DNA, for whom eMLST analysis was successfully performed, had a lower rate of concurrent presence of amniotic fluid *U.* spp. DNA than those with PPROM (17% [9/53] vs. 59% [20/34];* p* = 0.0001; see Fig. 1). Amniotic fluid microbial features in pregnant women with PTL and PPROM regarding the presence or absence of cervical fluid *U.* spp. DNA are listed in Supplementary Table 4.

### U. parvum (cluster I) and U. urealyticum (cluster II) in cervical fluid

#### Pregnant women with PTL

*U. parvum* and *U. urealyticum* were detected in 92% (49/53) and 8% (4/53) of the samples, respectively. No differences were identified regarding demographic or clinical parameters between *U. parvum* and *U. urealyticum* except for a higher body mass index (BMI) in the *U. parvum* group (Table 1).

**Table 1:** Demographical and clinical characteristics of the pregnant women with preterm labor with intact membranes harboring *Ureaplasma* spp. DNA in the cervical fluid, in whom the expanded multilocus sequence-typing scheme was performed, regarding the clusters of cervical fluid *Ureaplasma* spp. DNA.

Characteristic	Cluster I (*U. parvum*) (n = 49)	Cluster II (*U. urealyticum*) (n = 4)	Exact *P-*value
Maternal age [years, median (IQR)]	28 (23–31)	26 (22–31)	0.74
Nulliparous [number (%)]	31 (63%)	1 (25%)	0.29
Smoking [number (%)]	11 (22%)	2 (50%)	0.25
Pre-pregnancy body mass index [kg/m^2^, median (IQR)]	25.0 (22.2–27.9)	20.5 (18.0–22.9)	**0.02**^**#**^
Gestational age at sampling [weeks + days, median (IQR)]	29 + 5 (25 + 4–32 + 0)	28 + 1 (25 + 0–32 + 3)	0.76
Gestational age at delivery [weeks + days, median (IQR)]	31 + 5 (27 + 3–35 + 2)	35 + 1 (29 + 6–37 + 5)	0.28
Latency from admission to delivery [days, median (IQR)]	4 (1–29)	42 (8–72)	0.25
Amount of cervical fluid *U.* spp. DNA [copies DNA/mL, median (IQR)]	2.4 × 10^5^ (1.3 × 10^4^–2.3 × 10^6^)	9.3 × 10^5^ (2.3 × 10^5^–1.0 × 10^7^)	0.38
Relative abundance of cervical fluid *U*. spp. DNA [%, median (IQR)]	0.7 (0.1–4.1)	16.8 (0.2–32.6)	0.18
Amniotic fluid IL-6 concentrations [pg/mL, median (IQR)]	4,000 (1054–23,212)	9711 (231–42,259)	0.73
Intra-amniotic infection [number (%)]	12 (25%)	1 (25%)	1.00
Sterile intra-amniotic inflammation [number (%)]	16 (33%)	1 (25%)	1.00
Without inflammation and microorganisms [number (%)]	21 (43%)	2 (50%)	1.00
CRP levels at admission [mg/L, median (IQR)]	7.1 (3.1–18.0)	12.5 (2.4–24.8)	0.97
WBC count at admission [× 10^9^ L, median (IQR)]	14.5 (11.9–16.9)	12.7 (4.9–23.2)	0.79
Administration of corticosteroids [number (%)]	43 (88%)	3 (75%)	0.44
Administration of antibiotics [number (%)]	31 (63%)	3 (75%)	1.00
Spontaneous vaginal delivery [number (%)]	38 (78%)	3 (75%)	1.00
Birth weight [grams, median (IQR)]	1810 (990–2350)	2035 (1048–3210)	0.57
Apgar score < 7; 5 min [number (%)]	6 (12%)	0 (0%)	1.00
Apgar score < 7; 10 min [number (%)]	5 (10%)	0 (0%)	1.00

CRP, C-reactive protein; IL, interleukin; IQR, interquartile range; WBC, white blood cells.

Continuous variables, presented as median (interquartile range), were compared using a nonparametric Mann–Whitney *U* test. Categorical variables, presented as number (%), were compared using Fisher’s exact test. Statistically significant results are marked in bold.

^#^The result does not remain statistically significant after applying the Bonferroni correction for multiple comparisons (adjusted significance threshold: *p* = 0.002).

#### Pregnant women with PPROM

*U. parvum* and *U. urealyticum* were detected in 82% (28/34) and 18% (6/34) of the samples, respectively. *U. parvum* was associated with a higher rate of nulliparity and a lower rate of colonization of the amniotic cavity than *U. urealyticum* (Table 2).

**Table 2:** Demographical and clinical characteristics of the pregnant women with preterm prelabor rupture of membranes harboring *Ureaplasma* spp. DNA in the cervical fluid, in whom expanded multilocus sequence-typing scheme was performed, regarding the clusters of cervical fluid *Ureaplasma* spp. DNA.

	Cluster I *(U. parvum*) (n = 28)	Cluster II *(U. urealyticum)* (n = 6)	Exact* P*-value
Maternal age [years, median (IQR)]	30 (24–34)	29 (25–35)	0.92
Nulliparous [number (%)]	18 (64%)	0 (0%)	**0.006**^**#**^
Smoking [number (%)]	9 (32%)	2 (33%)	1.00
Pre-pregnancy body mass index [kg/m^2^, median (IQR)]	25.1 (20.0–27.2)	25.0 (22.2–28.1)	0.85
Gestational age at sampling [weeks + days, median (IQR)]	33 + 1 (30 + 3–35 + 2)	33 + 1 (31 + 6–35 + 3)	0.30
Gestational age at delivery [weeks + days, median (IQR)]	33 + 4 (31 + 2–35 + 2)	34 + 2 (33 + 4–35 + 4)	0.27
Latency from PPROM to amniocentesis [hours, median (IQR)	4 (2–7)	5 (5–16)	0.38
Latency from PPROM to delivery [hours, median (IQR)]	45 (17–91)	59 (27–137)	0.59
Amount of cervical fluid *U.* spp. DNA [copies DNA/mL, median (IQR)]	7.0 × 10^4^ (4.3 × 10^3^–2.5 × 10^5)^	1.5 × 10^5^ (5.5 × 10^4^–2.5 × 10^5^)	0.26
Relative abundance of cervical fluid *U*. spp. DNA [%, median (IQR)]	0.6 (0.1–6.2)	2.4 (0.4–32.2)	0.34
Amniotic fluid IL-6 concentrations [pg/mL, median (IQR)]	2,157 (786–15,349)	1,141 (714–11,472)	0.46
Intra-amniotic infection [number (%)]	13 (46%)	1 (17%)	0.36
Sterile intra-amniotic inflammation [number (%)]	0 (0%)	0 (0%)	–
Colonization of the amniotic cavity [number (%)]	6 (21%)	4 (67%)	**0.05**^**#**^
Without inflammation and microorganisms [number (%)]	9 (32%)	1 (17%)	0.65
CRP levels at admission [mg/L, median (IQR)]	8.2 (3.2–19.6)	8.5 (4.6–16.3)	0.99
WBC count at admission [× 10^9^ L, median (IQR)]	12.4 (10.3–15.5)	13.6 (9.5–14.3)	0.89
Administration of corticosteroids [number (%)]	22 (79%)	4 (67%)	0.61
Administration of antibiotics [number (%)]	28 (100%)	6 (100%)	–
Spontaneous vaginal delivery [number (%)]	21 (75%)	4 (67%)	0.65
Cesarean section [number (%)]	6 (21%)	2 (33%)	0.61
Forceps/vacuumextraction delivery [number (%)]	1 (4%)	0 (0%)	1.00
Birth weight [grams, median (IQR)]	2050 (1,760–2,333)	2350 (2020–2738)	0.12
Apgar score < 7; 5 min [number (%)]	1 (4%)	0 (0%)	1.00
Apgar score < 7; 10 min [number (%)]	1 (4%)	0 (0%)	1.00

CRP, C-reactive protein; IL, interleukin; IQR, interquartile range; PPROM, preterm prelabor rupture of membranes; WBC, white blood cells.

Continuous variables, presented as median (interquartile range), were compared using a nonparametric Mann–Whitney *U* test. Categorical variables, presented as number (%), were compared using Fisher’s exact test. Statistically significant results are marked in bold.

^#^The result does not remain statistically significant after applying the Bonferroni correction for multiple comparisons (adjusted significance threshold: *p* = 0.002).

#### Comparison between pregnant women with PTL and PPROM

Pregnant women with PTL and PPROM showed no difference in the prevalence rates of *U. parvum* and *U. urealyticum* (*p* = 0.18).

### *Phylogenetic analysis and subdivision of cervical fluid U*.* spp. DNA*

#### Pregnant women with PTL

**Fig. 2:**
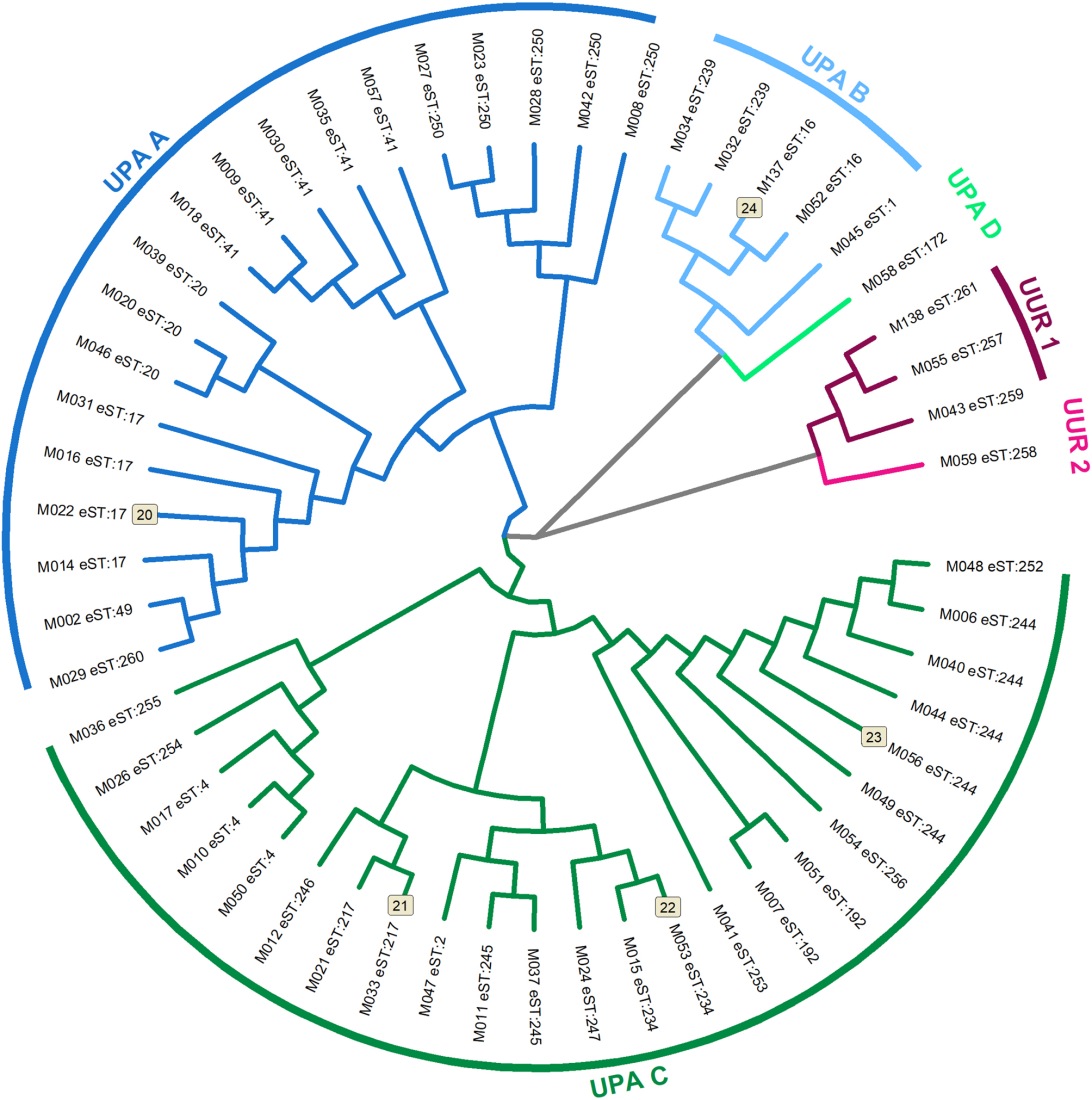
A neighbor-joining tree based on concatenated nucleotide sequences of the strains of *Ureaplasma* spp. DNA obtained from cervical from pregnant women with preterm labor with intact membranes. Two genetically significant clusters (I—*Ureaplasma parvum* and II—*Ureaplasma urealyticum*) and six subgroups (subgroups A [UPA A], B [UPA B], C [UPA C], and D [UPA)D in cluster I and subgroups 1 [UUR 1] and 2 [UUR 2] in cluster II) are depicted. The expanded sequence types are presented at the top of each branch. Cervical fluid *Ureaplasma* spp. DNA isolates for which paired amniotic fluid *Ureaplasma* spp. DNA isolates were available are marked with a number at the top of each branch (n = 5).

Among the pregnant women with PTL, 28 different eSTs of cervical fluid *U.* spp. DNA were revealed. The most frequent was #244, #41, and #250 in 11% (n = 6), 9% (n = 5), and 9% (n = 5) of the aliquots, respectively. To determine the evolutionary relationships among the 28 eSTs of *U.* spp. DNA, a neighbor-joining tree was constructed based on the sequences of the six loci involved in the eMLST (Fig. 2). Based on the genetic relationships, cluster I (*U. parvum*) was subclassified into four (A, 36% [n = 19]; B, 9% [n = 5]; C, 45% [n = 24]; and D, 2% [n = 1]) and cluster II (*U. urealyticum*) into two (1, 6% [n = 3]; and 2, 2% [n = 1]) subgroups previously described^16^.

No differences in demographic and clinical parameters, except for BMI, were found among the subgroups of *U.* spp. DNA (subgroups D and 2 were not considered in the analyses because of the small sample size [n = 1 in both]) (Table 3).

**Table 3:** Demographical and clinical characteristics of the pregnant women with preterm labor with intact membranes harboring *Ureaplasma* spp. DNA in the cervical fluid, in whom the expanded multilocus sequence-typing scheme was performed, regarding the subgroups of *Ureaplasma* spp. DNA.

	UPA A (n = 19)	UPA B (n = 5)	UPA C (n = 24)	UUR 1 (n = 3)	*P*-value
Maternal age [years, median (IQR)]	28 (22–31)	25 (22–29)	28 (24–31)	23 (21–28)	0.65
Primiparous [number (%)]	11 (58%)	4 (80%)	15 (63%)	1 (33%)	0.66
Smoking [number (%)]	2 (11%)	2 (40%)	7 (29%)	2 (67%)	0.09
Pre-pregnancy body mass index [kg/m^2^, median (IQR)]	23.7 (20.9–27.8)	24.6 (20.8–25.1)	26.1 (23.3–29.2)	18.4 (17.8–22.6)	**0.04**^**#**^
Gestational age at sampling [weeks + days, median (IQR)]	29 + 2 (27 + 5–31 + 2)	24 + 6 (24 + 1–30 + 5)	30 + 6 (24 + 3–34 + 1)	29 + 2 (26 + 2–33 + 3)	0.61
Gestational age at delivery [weeks + days, median (IQR)]	33 + 6 (30 + 4–36 + 5)	31 + 0 (25 + 6–33 + 2)	31 + 2 (25 + 6–34 + 5)	36 + 6 (33 + 3–38 + 0)	0.06
Latency from admission do delivery [hours, median (IQR)]	2 (0–54)	16 (3–41)	4 (1–10)	53 (0–78)	0.63
Amount of cervical fluid *U.* spp. DNA [copies DNA/mL, median (IQR)]	2.3 × 10^5^ (2.4 × 10^4^–1.3 × 10^6^)	1.2 × 10^6^ (5.1 × 10^4^–2.3 × 10^6^)	6.4 × 10^5^ (9.8 × 10^3^–2.6 × 10^6^)	1.0 × 10^6^ (8.5 × 10^5^–1.3 × 10^7^)	0.58
Relative abundance of cervical fluid *U*. spp. DNA [%, median (IQR)]	0.8 (0.3–6.5)	0.4 (0.1–7.0)	0.7 (0.1–4.3)	16.8 (0.2–32.6)	0.78
Amniotic fluid IL-6 concentrations [pg/mL, median (IQR)]	1434 (407–12,353)	4000 (1,559–27,025)	5985 (1,269–45,972)	387 (179–50,000)	0.33
Intra-amniotic infection [number (%)]	3 (16%)	1 (20%)	8 (33%)	1 (33%)	0.54
Sterile intra-amniotic inflammation [number (%)]	5 (26%)	2 (40%)	8 (33%)	0 (0%)	0.72
Without inflammation/microorganisms [number (%)]	11 (58%)	2 (40%)	8 (33%)	2 (67%)	0.36
CRP levels at admission [mg/L, median (IQR)]	7.1 (2.9–17.0)	4.9 (3.6–16.8)	7.1 (3.4–20.1)	20.6 (4.3–26.2)	0.84
WBC count at admission [× 10^9^ L, median (IQR)]	14.5 (12.5–18.2)	10.9 (7.3–14.15)	14.9 (12.8–16.5)	16.7 (3.6–25.4)	0.26
Administration of corticosteroids [number (%)]	16 (84%)	5 (100%)	24 (100%)	2 (67%)	0.06
Administration of antibiotics [number (%)]	12 (63%)	3 (60%)	16 (67%)	2 (67%)	1.00
Spontaneous vaginal delivery [number (%)]	14 (74%)	3 (60%)	20 (83%)	2 (67%)	0.52
Birth weight [grams, median (IQR)]	2060 (1380–2690)	1410 (825–2290)	1680 (799–2283)	2490 (1580–3450)	0.11
Apgar score < 7; 5 min [number (%)]	0 (0%)	1 (20%)	4 (17%)	0 (0%)	0.18
Apgar score < 7; 10 min [number (%)]	0 (0%)	0 (0%)	4 (17%)	0 (0%)	0.27

CRP, C-reactive protein; IL, interleukin; IQR, interquartile range; PPROM, preterm prelabor rupture of membranes; UPA A, *U. parvum* subgroup A; UPA B, *U. parvum* subgroup B; UPA C, *U. parvum* subgroup C; UUR 1, *U. urealyticum* subgroup 1; WBC, white blood cells.

The *U. parvum* subgroup D and *U. urealyticum* subgroup 2 were not included in the analyses due to very low sample size (both n = 1).

Continuous variables, presented as median (interquartile range), were compared using a nonparametric Kruskal–Wallis *H* test. Categorical variables, presented as number (%), were compared using Chi-square test. Statistically significant results are marked in bold.

^#^The result does not remain statistically significant after applying the Bonferroni correction for multiple comparisons (adjusted significance threshold: *p* = 0.002).

#### Pregnant women with PPROM

**Fig. 3:**
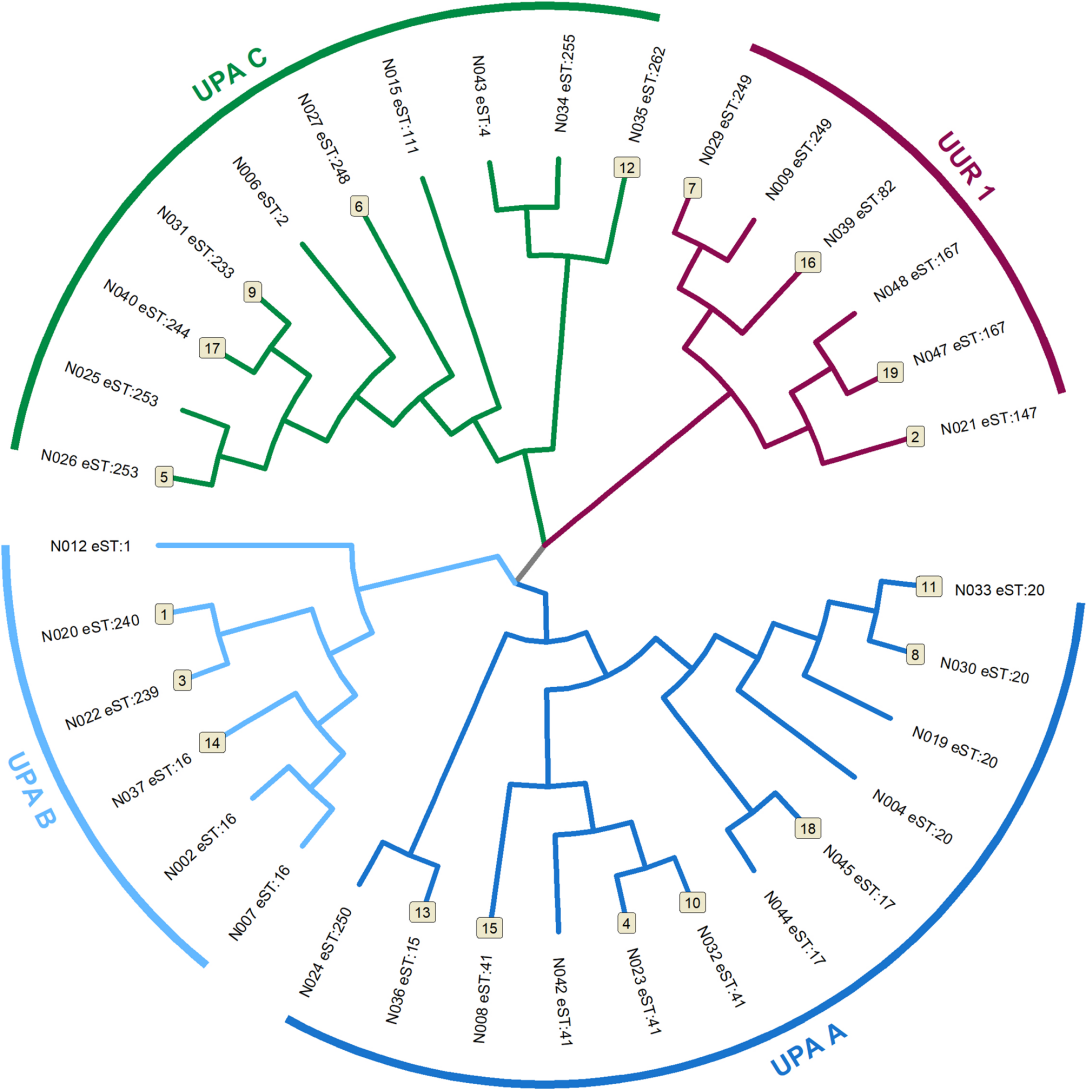
A neighbor-joining tree based on concatenated nucleotide sequences of the strains of *Ureaplasma* spp. DNA obtained from cervical pregnant women with preterm prelabor rupture of membranes. Two genetically significant clusters (I—*Ureaplasma parvum* and II—*Ureaplasma urealyticum*) and four subgroups (subgroups A [UPA A], B [UPA B], and C [UPA C] in cluster I and subgroup 1[UUR 1] in cluster II) are depicted. The expanded sequence types are presented at the top of each branch. Cervical fluid *Ureaplasma* spp. DNA isolates for which paired amniotic fluid *Ureaplasma* spp. DNA isolates were available are marked with a number at the top of each branch (n = 19).

Twenty-two different eSTs were identified among the aliquots of cervical fluid *U.* spp. DNA. The most frequent eSTs in the cervical fluid *U.* spp. DNA were as follows: #41, #20, and #16 in 12% (n = 4), 12% (n = 4), and 9% (n = 3) of the pregnant women, respectively. A neighbor-joining tree was created to characterize the evolutionary relationship between the identified 22 eSTs of *U.* spp. DNA (Fig. 3). Cluster I (*U. parvum*) was further subclassified into three subgroups (A, 35% [n = 12]; B, 17% [n = 6]; C, 29% [n = 10]) previously described^16^. Cluster II (*U. urealyticum*) consisted of only one subgroup previously described^16^. No differences in demographic or clinical parameters, except for the rate of nulliparity, were observed among the subgroups of *U.* spp. DNA (Table 4).

**Table 4:** Demographical and clinical characteristics of the pregnant women with preterm prelabor rupture of membranes harboring *Ureaplasma* spp. DNA in the cervical fluid, in whom the expanded multilocus sequence-typing scheme was performed, regarding *Ureaplasma* spp. DNA subgroups.

	UPA A (n = 12)	UPA B (n = 6)	UPA C (n = 10)	UUR 1 (n = 6)	*P*-value
Maternal age [years, median (IQR)]	27 (22–32)	35 (26–37)	30 (27–34)	29 (25–35)	0.37
Nulliparous [number (%)]	7 (58%)	5 (83%)	6 (60%)	0 (0%)	**0.03**^**#**^
Smoking [number (%)]	3 (25%)	1 (17%)	5 (50%)	2 (33%)	0.55
Pre-pregnancy body mass index [kg/m^2^, median (IQR)]	25.9 (21.2–27.4)	23.8 (18.8–27.5)	24.3 (19.8–27.4)	25.0 (22.2–28.1)	0.84
Gestational age at sampling [weeks + days, median (IQR)]	32 + 2 (28 + 4–34 + 2)	33 + 4 (30 + 6–35 + 5)	33 + 2 (29 + 6–35 + 3)	33 + 1 (31 + 6–35 + 3)	0.50
Gestational age at delivery [weeks + days, median (IQR)]	33 + 1 (31 + 4–34 + 3)	33 + 5 (31 + 2–35 + 5)	33 + 4 (31 + 2–35 + 5)	34 + 2 (33 + 4–35 + 4)	0.56
Latency from PPROM to amniocentesis [hours, median (IQR)]	5 (3–7)	5 (2–9)	3 (1–7)	5 (5–16)	0.38
Latency from PPROM to delivery [hours, median (IQR)]	69 (11–110)	34 (20–79)	45 (18–75)	59 (27–137)	0.86
Amount of cervical fluid *U.* spp. DNA [copies DNA/mL, median (IQR)]	5.0 × 10^4^ (4.3 × 10^3^–2.5 × 10^5^)	9.0 × 10^3^ (5.3 × 10^3^–1.6 × 10^6^)	1.0 × 10^5^ (7.8 × 10^3^–3.3 × 10^5^)	1.5 × 10^5^ (5.5 × 10^4^–2.5 × 10^5^)	0.95
Relative abundance of cervical fluid *U*. spp. DNA [%, median (IQR)]	1.2 (0.1–9.7)	0.6 (0.4–4.5)	2.9 (1.0–27.3)	2.4 (0.4–32.2)	0.98
Amniotic fluid IL-6 concentrations [pg/mL, median (IQR)]	3540 (1,075–45,370)	3381 (766–15,755)	1618 (624–13,120)	1141 (714–11,472)	0.70
Intra-amniotic infection [number (%)]	6 (50%)	3 (50%)	4 (40%)	1 (17%)	0.62
Sterile intra-amniotic inflammation [number (%)]	0 (0%)	0 (0%)	0 (0%)	0 (0%)	–
Colonization of the amniotic cavity [number (%)]	3 (25%)	0 (0%)	3 (30%)	4 (67%)	0.10
Without inflammation/microorganisms [number (%)]	3 (25%)	3 (50%)	3 (30%)	1 (17%)	0.69
CRP levels at admission [mg/L, median (IQR)]	7.8 (2.4–20.0)	7.6 (4.9–21.7)	8.4 (2.9–23.1)	8.5 (4.6–16.3)	1.00
WBC count at admission [× 10^9^ L, median (IQR)]	12.7 (10.9–15.8)	11.0 (9.2–14.5)	13.7 (9.9–15.6)	13.6 (9.5–14.3)	0.80
Administration of corticosteroids [number (%)]	11 (92%)	4 (67%)	7 (70%)	4 (67%)	0.43
Administration of antibiotics [number (%)]	12 (100%)	6 (100%)	10 (100%)	6 (100%)	–
Spontaneous vaginal delivery [number (%)]	9 (75%)	4 (67%)	8 (80%)	4 (67%)	0.90
Cesarean section [number (%)]	3 (25%)	1 (17%)	2 (20%)	2 (33%)	0.95
Forceps/vacuumextraction delivery [number (%)]	0 (0%)	1 (17%)	0 (0%)	0 (0%)	0.35
Birth weight [grams, median (IQR)]	1975 (1843–2328)	2240 (1600–2508)	1975 (1515–2243)	2350 (2020–2738)	0.34
Apgar score < 7; 5 min [number (%)]	0 (0%)	0 (0%)	1 (10%)	0 (0%)	0.65
Apgar score < 7; 10 min [number (%)]	0 (0%)	0 (0%)	1 (10%)	0 (0%)	0.65

CRP, C-reactive protein; IL, interleukin; IQR, interquartile range; PPROM, preterm prelabor rupture of membranes; UPA A, *U. parvum* subgroup A; UPA B, *U. parvum* subgroup B; UPA C, *U. parvum* subgroup C; UUR 1, *U. urealyticum* subgroup 1; WBC, white blood cells.

Continuous variables, presented as median (interquartile range), were compared using a nonparametric Kruskal–Wallis *H* test. Categorical variables, presented as number (%), were compared using chi-square test. Statistically significant results are marked in bold.

^#^The result does not remain statistically significant after applying the Bonferroni correction for multiple comparisons (adjusted significance threshold: *p* = 0.002).

#### Comparison between pregnant women with PTL and PPROM

Twelve eSTs of cervical fluid *U.* spp. DNA (#1, #2, #4, #16, #17, #20, #41, #239, #244, #250, #253, and #255) were identified in both PTL and PPROM. Sixteen eSTs (#49, #172, #192, #217, #234, #245-247, #252, #254, #256-261) were found only in PTL, whereas 10 eSTs (#15, #82, #111, #147, #167, #233, #240, #248, #249, #262) were identified only in PPROM.

No differences were observed between pregnant women with PTL and PPROM regarding the proportions of the subgroups of cervical fluid *U.* spp. DNA (A, *p* = 1.00; B, *p* = 0.33; C, *p* = 0.18; D, *p* = 1.00; 1, *p* = 0.15; and 2, *p* = 1.00). Similarly, no differences were found in the rates of the most common eSTs in the cervical fluid *U.* spp. DNA between pregnant women with PTL and PPROM (#16, *p* = 0.37; #20, *p* = 0.43; #41, *p* = 0.73; #244, *p* = 0.24; and #250, *p* = 0.40).

### Concurrent presence of amniotic fluid U. spp. DNA

#### Pregnant women with PTL

Aliquots of amniotic fluid* U*. spp. DNA for the eMLST analyses were available for only five of nine pregnant women with concurrent amniotic fluid *U*. spp. DNA. eMLST analysis was successful in all cases. No differences were observed in the proportions of clusters and subgroups of cervical fluid from *U.* spp. DNA identified in pregnant women with and without concurent amniotic fluid *U*. spp. DNA (Table 5).

**Table 5:** Demographical and clinical characteristics of the pregnant women with preterm labor with intact membranes harboring *Ureaplasma* spp. DNA in the cervical fluid, in whom the expanded multilocus sequence-typing scheme was performed, regarding the presence or absence of *Ureaplasma* spp. DNA in amniotic fluid.

Characteristic	The presence of amniotic fluid *Ureaplasma* spp. DNA (n = 5)	The absence of amniotic fluid *Ureaplasma* spp. DNA (n = 44)	*P-*value
Maternal age [years, median (IQR)]	29 (26–34)	28 (22–31)	0.33
Primiparous [number (%)]	2 (40%)	28 (64%)	0.34
Smoking [number (%)]	2 (40%)	10 (23%)	0.58
Pre-pregnancy body mass index [kg/m^2^, median (IQR)]	29.2 (22.2–36.7)	23.8 (22.0–27.8	0.17
Gestational age at sampling [weeks + days, median (IQR)]	27 + 2 (23 + 2–31 + 3)	29 + 5 (25 + 6–32 + 2)	0.25
Gestational age at delivery [weeks + days, median (IQR)]	31 + 0 (26 + 0–34 + 1)	32 + 3 (26 + 4–35 + 5)	0.53
Latency from admission to delivery [days, median (IQR)]	28 (0–48)	4 (1–30)	0.90
Amount of cervical fluid U. spp. DNA [copies DNA/mL, median (IQR)]	9.3 × 10^3^ (2.0 × 10^3^–5.2 × 10^4^)	5.9 × 10^5^ (4.3 × 10^4^–2.3 × 10^6^)	**0.006**^**#**^
Relative abundance of cervical fluid U. spp. DNA [%, median (IQR)]	0.05 (0.01–0.72)	0.82 (0.21–6.30)	**0.002**^**#**^
Amniotic fluid IL-6 concentrations [pg/mL, median (IQR)]	50,000 (31,992–50,000)	2499 (542–10,419)	**0.0004**
Intra-amniotic infection [number (%)]	5 (100%)	4 (9%)	**< 0.0001**
Sterile intra-amniotic inflammation [number (%)]	0 (0%)	17 (39%)	0.15
Without inflammation/microorganisms [number (%)]	0 (0%)	23 (52%)	0.05
Cervical fluid *Ureaplasma* spp. DNA – cluster I	5 (100%)	41 (93%)	1.00
Cervical fluid *Ureaplasma* spp. DNA – cluster II	0 (0%)	3 (7%)	1.00
Cervical fluid *Ureaplasma* spp. DNA subgroup UPA-A [number (%)]	1 (20%)	17 (39%)	0.64
Cervical fluid *Ureaplasma* spp. DNA subgroup UPA-B [number (%)]	1 (20%)	4 (9%)	0.43
Cervical fluid *Ureaplasma* spp. DNA subgroup UPA-C [number (%)]	3 (60%)	19 (43%)	0.65
Cervical fluid *Ureaplasma* spp. DNA subgroup UPA-D [number (%)]	0 (0%)	1 (2%)	1.00
Cervical fluid *Ureaplasma* spp. DNA subgroup UUR-1 [number (%)]	0 (0%)	2 (5%)	1.00
Cervical fluid *Ureaplasma* spp. DNA subgroup UUR-2 [number (%)]	0 (0%)	1 (2%)	1.00
CRP levels at admission [mg/L, median (IQR)]	10.3 (5.5–29.7)	6.6 (2.7–16.8)	0.31
WBC count at admission [× 10^9^ L, median (IQR)]	14.5 (8.8–18.6)	13.8 (11.0–16.5)	0.97
Administration of corticosteroids [number (%)]	5 (100%)	38 (86%)	1.00
Administration of antibiotics [number (%)]	4 (80%)	27 (61%)	0.64
Spontaneous vaginal delivery [number (%)]	3 (60%)	36 (82%)	0.27
Birth weight [grams, median (IQR)]	1410 (985–2105)	1895 (1010–2488)	0.34
Apgar score < 7; 5 min [number (%)]	0 (0%)	6 (14%)	1.00
Apgar score < 7; 10 min [number (%)]	0 (0%)	5 (11%)	1.00

CRP, C-reactive protein; IL, interleukin; IQR, interquartile range; WBC, white blood cells.

Continuous variables, presented as median (interquartile range), were compared using a nonparametric Mann–Whitney *U* test. Categorical variables, presented as number (%), were compared using Fisher’s exact test. Statistically significant results are marked in bold.

^#^The result does not remain statistically significant after applying the Bonferroni correction for multiple comparisons (adjusted significance threshold: *p* = 0.002).

**Fig. 4:**
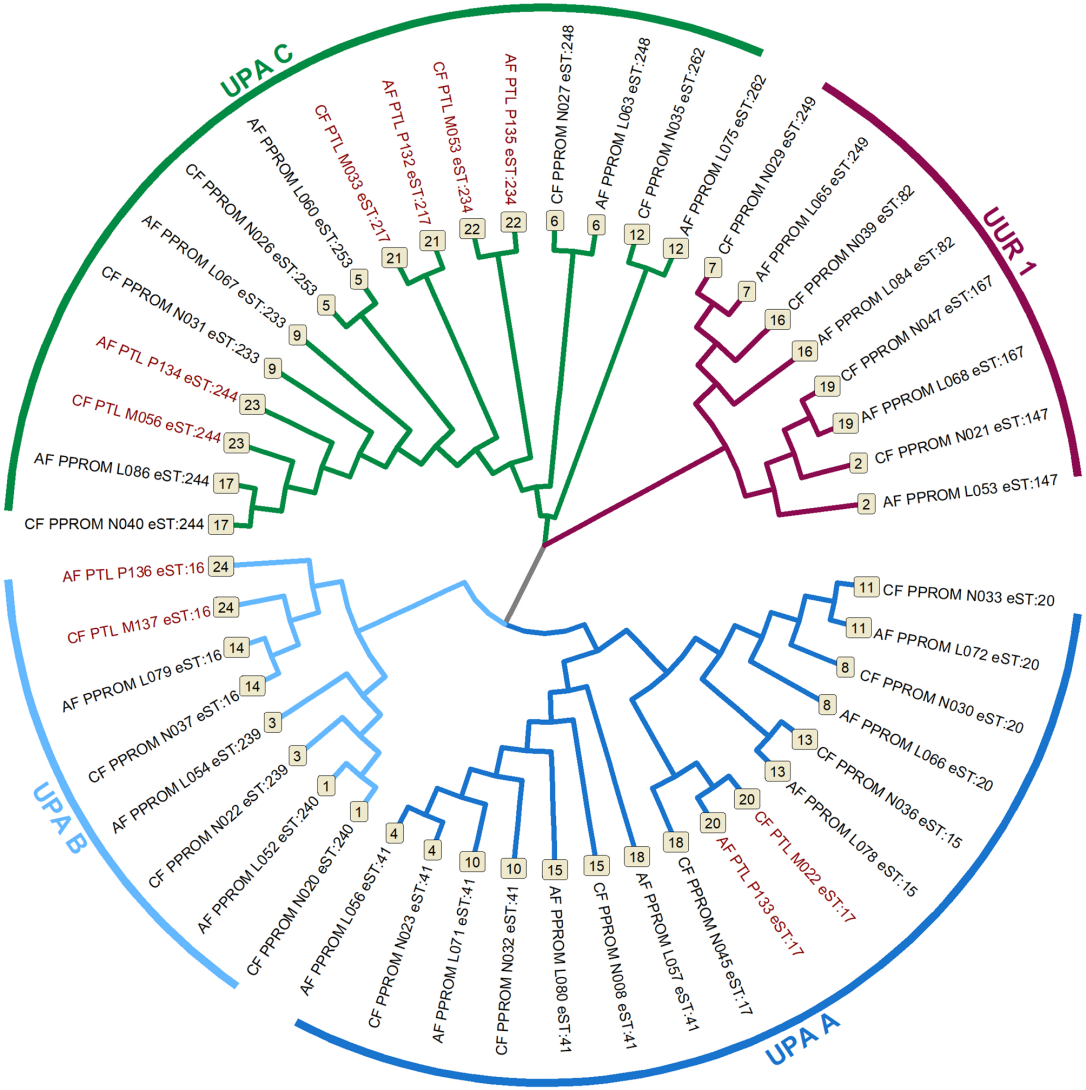
A neighbor-joining tree based on concatenated nucleotide sequences of the strains of *Ureaplasma* spp. DNA obtained from paired amniotic and cervical fluids in pregnant women with preterm labor with intact membranes and preterm prelabor rupture of membranes. Two genetically significant clusters (I—*Ureaplasma parvum* and II—*Ureaplasma urealyticum*) and four subgroups (subgroups A [UPA A], B [UPA B], and C [UPA C] in cluster I and subgroup 1 [UUR 1] in cluster II) are depicted. The expanded sequence types and phenotype of spontaneous preterm delivery are presented at the top of each branch. Pairs are marked with the same number at the top of the branch. Pairs from preterm labor with intact membranes and preterm prelabor rupture of membranes are marked in red and black, respectively.

Concordance between amniotic and cervical fluid eSTs of *U.* spp. DNA was detected in 100% (5/5) of the pairs (Supplementary Table 5). Figure 4 shows a neighbor-joining tree *of U.* spp. DNA from the paired samples.

### Pregnant women with PPROM

eMLST analysis of amniotic fluid *U.* spp. DNA was performed in our previous study, in which aliquots of amniotic fluid *U.* spp. DNA were available for 19 of 20 pregnant women with the concurrent presence of amniotic fluid *U.* spp. DNA^18^. The eST results were available for 19 pairs of cervical and amniotic fluid *U.* spp. DNA (Supplementary Table 6).

No differences were observed in the proportions of clusters and subgroups of cervical fluid *U.* spp. DNA detected in amniotic fluid with and without *U.* spp. DNA (Table 6). Pregnant women with *U*. spp. DNA in the amniotic fluid had a higher relative abundance of *U.* spp. DNA in the cervical fluid than those without *U.* spp. DNA (Table 6).

**Table 6:** Demographical and clinical characteristics of the pregnant women with preterm prelabor rupture of membranes harboring *Ureaplasma* spp. DNA in the cervical fluid, in whom the expanded multilocus sequence-typing scheme was performed, regarding the presence or absence of *Ureaplasma* spp. DNA in amniotic fluid.

Characteristic	The presence of amniotic fluid *Ureaplasma* spp. DNA (n = 19)	The absence of amniotic fluid *Ureaplasma* spp. DNA (n = 14)	*P-*value
Maternal age [years, median (IQR)]	32 (27–36)	27 (24–31)	0.12
Primiparous [number (%)]	9 (47%)	9 (64%)	0.48
Smoking [number (%)]	3 (16%)	7 (50%)	0.06
Pre-pregnancy body mass index [kg/m^2^, median (IQR)]	25.2 (20.2–27.0)	25.7 (22.6–28.9)	0.42
Gestational age at sampling [weeks + days, median (IQR)]	32 + 4 (30 + 2–35 + 3)	33 + 3 (32 + 4–34 + 4)	0.53
Gestational age at delivery [weeks + days, median (IQR)]	33 + 0 (31 + 1–35 + 3)	34 + 0 (32 + 5–34 + 5)	0.65
Latency from PPROM to amniocentesis [hours, median (IQR)]	6 (3–9)	4 (2–5)	0.23
Latency from PPROM to delivery [hours, median (IQR)]	64 (17–101)	24 (17–80)	0.48
Amount of cervical fluid *U.* spp. DNA [copies DNA/mL, median (IQR)]	1.0 × 10^5^ (7.0 × 10^4^–8.0 × 10^5^)	9.0 × 10^3^ (2.0 × 10^3^–1.0 × 10^5^)	**0.006**^**#**^
Relative abundance of cervical fluid *U*. spp. DNA [%, median (IQR)]	3.7 (2.2–38.5)	0.1 (0.0–0.7)	**0.0005**
Amniotic fluid IL-6 concentrations [pg/mL, median (IQR)]	5,000 (1,425–16,463)	845 (493–14,012)	**0.03**^**#**^
Intra-amniotic infection [number (%)]	11 (58%)	3 (21%)	0.07
Sterile intra-amniotic inflammation [number (%)]	0 (0%)	0 (0%)	–
Colonization of the amniotic cavity [number (%)]	8 (42%)	2 (14%)	0.13
Without inflammation/microorganisms [number (%)]	0 (0%)	9 (64%)	**< 0.0001**
Cervical fluid *Ureaplasma* spp. DNA – cluster I	15 (79%)	12 (86%)	1.00
Cervical fluid *Ureaplasma* spp. DNA – cluster II	4 (21%)	2 (14%)	1.00
Cervical fluid *Ureaplasma* spp. DNA subgroup UPA-A [number (%)]	7 (37%)	5 (36%)	1.00
Cervical fluid *Ureaplasma* spp. DNA subgroup UPA-B [number (%)]	3 (16%)	3 (21%)	1.00
Cervical fluid *Ureaplasma* spp. DNA subgroup UPA-C [number (%)]	5 (26%)	4 (29%)	1.00
Cervical fluid *Ureaplasma* spp. DNA subgroup UUR-1 [number (%)]	4 (21%)	2 (14%)	1.00
CRP levels at admission [mg/L, median (IQR)]	12.5 (3.3–21.9)	7.5 (3.5–11.4)	0.29
WBC count at admission [× 10^9^ L, median (IQR)]	12.2 (6.8–14.8)	13.0 (10.8–16.0)	0.66
Administration of corticosteroids [number (%)]	15 (79%)	11 (79%)	1.00
Administration of antibiotics [number (%)]	19 (100%)	14 (100%)	–
Spontaneous vaginal delivery [number (%)]	14 (74%)	10 (71%)	1.00
Cesarean section [number (%)]	5 (26%)	3 (21%)	1.00
Forceps/vacuumextraction delivery [number (%)]	0 (0%)	1 (7%)	0.42
Birth weight [grams, median (IQR)]	1940 (1620–2340)	2105 (1903–2443)	0.53
Apgar score < 7; 5 min [number (%)]	1 (5%)	0 (0%)	1.00
Apgar score < 7; 10 min [number (%)]	1 (5%)	0 (0%)	1.00

CRP, C-reactive protein; IL, interleukin; IQR, interquartile range; PPROM, preterm prelabor rupture of membranes; WBC, white blood cells.

Continuous variables, presented as median (interquartile range), were compared using a nonparametric Mann–Whitney *U* test. Categorical variables, presented as number (%), were compared using Fisher’s exact test. Statistically significant results are marked in bold.

^#^The result does not remain statistically significant after applying the Bonferroni correction for multiple comparisons (adjusted significance threshold: *p* = 0.002).

Concordance between cervical and amniotic fluid eSTs of *U*. spp. DNA was found in 95% (18/19) of the pairs with a discordant pair: cervical fluid—#17, amniotic fluid—#41. Figure 4 shows a neighbor-joining tree of *U.* spp. DNA from the paired samples.

## Discussion

*U.* spp. is closely associated with intra-amniotic complications in pregnant women with spontaneous preterm delivery^8,11,12,23,24^. The principal findings of this study regarding the cervical fluid *U.* spp. DNA from pregnant women with both phenotypes of spontaneous preterm delivery are as follows: (i) the most common eST of cervical fluid *U.* spp. was #41; (ii) no major differences in demographic and clinical parameters were found between the pregnant women with cervical fluid *U. parvum* and *U. urealyticum* DNA, except a higher rate of colonization of the amniotic cavity found in those with PPROM harboring cervical fluid *U. urealyticum*; (iii) no major differences in demographic and clinical parameters were found among women with different subgroups of cervical fluid *U.* spp.; (iv) a concordance in eSTs between paired samples of amniotic and cervical fluids *U*. spp. reached 100% and 95% in PTL and PPROM, respectively; (v) no differences in the frequency of the clusters (*U. parvum* and *U. urealyticum*) and subgroups of cervical fluid *U*. spp. were identified between pregnant women with and without concomitant presence of amniotic fluid *U.* spp.; and (vi) no differences in the rates of clusters, subgroups, and the most common eSTs of cervical fluid *U.* spp. were found between the pregnant women with PTL and PPROM.

The employment of the eMLST scheme in genotyping *U.* spp. provides a unique opportunity to dissect more than 200 different eSTs^16,17,25,26^. In the present study, #41 was the most common eST of *U*. spp. DNA identified in the cervix of pregnant women with spontaneous preterm deliveries. This observation agrees with that of our previous study, which focused on the eMLST scheme of amniotic fluid *U.* spp. DNA from pregnancies complicated by PPROM, in which eST #41 was found to be the most frequent and accounted for 18% of all *U.* spp. DNA^18^. The same eST #41 has been identified as the most predominant among *U.* spp. isolated from the amniotic fluid, placenta, and umbilical cord of pregnant women with PPROM or prelabor rupture of membranes^17^. In addition, eST #41 was the most common *U.* spp. strain isolated from paired samples from couples with infertility (urine and semen specimens from male partners and cervical or vaginal swabs from female partners), as well as from the first void urine or semen of male patients with asthenozoospermia, azoospermia, urethritis, prostatitis, varicocele, or infertility^25,26^.

A pioneering study by Kim et al. reported that “parvo biovar” (*U. parvum*) of *U.* spp. in amniotic fluid is more frequent than “T960 biovar” (*U. urealyticum*) among pregnant women with spontaneous preterm delivery^27^. In addition, they showed that biovar diversity is not related to differences in pregnancy outcomes, as well as in the intensity of intra-amniotic inflammation^27^. The results from this present study agree with those of the abovementioned study; in pregnant women with PTL and PPROM, cervical fluid *U. parvum* was responsible for 93% and 82% of cervical fluid *U.* spp. DNA, respectively. Similarly, in pregnant women with PTL and PPROM, amniotic fluid *U. parvum* was responsible for 100% and 79% of amniotic fluid *U.* spp. DNA, respectively. Interestingly, pregnant women with PPROM with cervical fluid *U. urealyticum* DNA had a higher rate of colonization of the amniotic cavity than those with *U. parvum* (21% vs. 67%). Colonization of the amniotic cavity represents a specific form of intra-amniotic complication that is almost exclusively associated with PPROM beyond the gestational age of 31 weeks^9^. Based on the available evidence, colonization might be characterized by the presence of a lower microbial burden of microorganisms or their nucleic acids in the amniotic fluid that elicits a weak intra-amniotic inflammatory response that is not intense enough to pass the threshold concentration of IL-6 (or other markers used in clinics to determine intra-amniotic inflammation) in the amniotic fluid^9^. The demographic and clinical characteristics of pregnant women with amniotic cavity colonization more closely resemble those of pregnant women with no detectable microorganisms or inflammation in the amniotic fluid than those with intra-amniotic infection or sterile intra-amniotic inflammation^9^. Nevertheless, when compared to pregnant women with a sterile amniotic environment, those with colonization exhibit higher concentrations of IL-6 and increased rates of acute inflammatory lesions in the placenta and funisitis^9^. These findings underscore that colonization of the amniotic cavity is not equivalent to a sterile intra-amniotic state and should not be interpreted as the absence of microorganisms or inflammation. Although observation that women with PPROM with cervical fluid *U. urealyticum* DNA had a higher rate of colonization of the amniotic cavity than those with *U. parvum* is of clinical interest, it should be interpreted with caution because of the small sample size of pregnant women with PPROM harboring cervical fluid *U. urealyticum* (n = 6). Taken together, *U. parvum* is by far the most prevalent biovar of *U.* spp. implicated in spontaneous preterm delivery; however, the rate of intra-amniotic inflammatory complications related to this biovar does not differ from those of *U. urealyticum*, except for a lower rate of colonization of the amniotic cavity in PPROM pregnancies.

Six different subgroups of *U.* spp. have been described so far: four for *U. parvum* (A, B, C, and D) and two for *U. urealyticum* (1 and 2)^15,16^. Pregnant women with PTL and PPROM were divided into cervical fluid *U*. spp. DNA subgroups, with no differences in the rates of intra-amniotic complications among the subgroups. This observation supports the theory that *U.* spp. genotypes might not be fundamentally “pathogenic” or “commensal”; rather, other factors such as their microbial burden, host response, or the presence of other bacteria might instead contribute to the development of intra-amniotic complications and adverse outcomes associated with their presence in the cervix.

Although several pathways have been proposed by which microorganisms can reach the amniotic cavity, ascension from the vagina and cervix is the most common one^28^. Nevertheless, for a long time, evidence supporting this pathway relied on the indirect observations that bacteria identified in amniotic fluid are typical microorganisms of the cervical/vaginal microbiota (e.g*. U.* spp., *Mycoplasma hominis*, *Streptococcus* spp., and *Sneathia* spp.)^28^. In 2019, Romero et al. provided the first direct observation that the ascent of microorganisms is the primary cause of intra-amniotic infection through a sequencing-based comparison of the microbial compositions of paired amniotic and vaginal fluid samples from pregnancies complicated by intra-amniotic infection^28^. This study further extends the direct evidence of the level of intra-amniotic infection and colonization of the amniotic cavity caused by *U.* spp. by confirming the concordance of eSTs of *U.* spp. obtained from paired amniotic and cervical fluids in pregnant women with PTL and PPROM. The discordance in the eST of *U.* spp. DNA between cervical and amniotic fluid samples was detected only in one woman with PPROM (cervical fluid—eST #17 and amniotic fluid eST—#4). The differences between these two eSTs were in two genes, *rpl22* (allele 1 in eST#17 and allele 2 in eST#41) and *ureG* (allele 2 in eST#17 and allele 3 in eST#41), which were relatively small. Similar discordances in the eSTs of *U.* spp. between paired samples were described previously by Kong et al. (in six out of 93 maternal/fetal pairs of amniotic fluid, umbilical cord, and placenta samples)^17^. The discordance in the STs of cervical and amniotic fluid *U*. spp. might be due to a mixture of *U.* spp. in a cervical fluid sample, from which only one strain was isolated and subsequently sequenced in eMLST analysis. However, we cannot exclude horizontal gene transfer, described previously in *U. urealyticum* and U. *parvum*^29,30^.

When both phenotypes of spontaneous preterm delivery (PTL and PPROM) were compared regarding the rates of clusters, subgroups, and the most common eST of cervical fluid *U.* spp., no substantial differences between phenotypes were identified. Recent in-vitro and in-vivo models of ascending infection have shown just a milder inflammatory response in maternal–fetal tissues caused by *U. parvum*^14,31–33^. However, in combination with other pathogenic organisms (e.g., *Escherichia coli, Gardnerella vaginalis*), U. *parvum* is much more pro-inflammatory^31–33^. Thus, the microbial burden and combination of microbes may determine the outcome. These observations, combined with the previously mentioned results, further support the hypothesis that it is not a specific genotype or strain of *U.* spp. that contributes to pathological outcomes but rather the presence of *U.* spp. along with other factors.

The main strength of this study is the comprehensive phenotyping of the pregnant women, dissecting both clinical phenotypes of spontaneous preterm delivery (PTL and PPROM). Second, the availability of paired cervical and amniotic fluid samples from both phenotypes of spontaneous preterm delivery makes this study unique. Finally, a more extensive eMLST scheme assessing six genes (including two putative virulence loci), rather than an MLST scheme assessing just four housekeeping genes of *U.* spp., was employed in this study.

However, the main limitation of this study is that cervical fluid samples from pregnant women with PPROM might be contaminated with amniotic fluid, which should be taken into consideration. In pregnant women with PPROM, a physiological barrier separating the cervical and intra-amniotic compartments is absent. Therefore, amniotic fluid leaking through the cervix is an unavoidable confounding factor in this subset of spontaneous preterm deliveries. Thus, the cervical fluid *U.* spp. DNA from pregnant women with PPROM may originate in part from the amniotic fluid, which might explain why women with PPROM who harbored amniotic fluid *U.* spp. DNA had a higher microbial load and relative abundance of cervical fluid *U.* spp. DNA than those without concomitant amniotic fluid *U.* spp. DNA. Interestingly, pregnant women with PTL had the opposite findings: those with concurent amniotic fluid *U.* spp. had a lower microbial load and relative abundance of cervical fluid *U.* spp. DNA than those without concurent amniotic fluid *U.* spp infection. DNA. Nevertheless, the latter observation should be interpreted with caution because of the limited sample size (n = 5). Next, given the observational nature of this exploratory genotyping study and the relatively rare availability of paired cervical and amniotic fluid samples**,** we did not perform an a priori power calculation. Finally, the homogeneity of the cohort of pregnant women (white pregnant women from the eastern part of the Czech Republic) might prevent the generalization of the results of this study to women with spontaneous preterm delivery of different races or from different regions.

In conclusion, cervical fluid *U.* spp. DNA obtained from pregnant women with spontaneous preterm delivery comprised 38 different genotypes. No distinct genotype, subgroup, or cluster of cervical fluid *U* spp DNA was identified as specifically associated with PTL or PPROM as well as with the ascension of *U.* spp. in the amniotic cavity in pregnant women with spontaneous preterm delivery.

## Methods

This nested retrospective cohort study included singleton pregnancies complicated by PPROM and PTL, admitted and treated at the Department of Obstetrics and Gynecology, University Hospital Hradec Kralove, in whom the presence of *U.* spp. DNA in the cervical fluid was previously assessed^11,12^. This study enrolled subsets of pregnant women with PTL with gestational ages between 22 + 0 and 34 + 6 weeks (admitted between January 2019 and February 2021) and pregnant women with PPROM with gestational ages between 24 + 0 and 34 + 6 weeks (admitted between January 2017 and August 2019). The inclusion and exclusion criteria for women with PTL and PPROM have been previously described^11,12^. In all included pregnancies, gestational age was determined by ultrasound measurement of crown-rump length during the first-trimester screening for chromosomal abnormalities between gestational ages 11 + 3 and 13 + 6 weeks.

PTL was diagnosed as the presence of regular uterine contractions (at least two contractions every 10 min) along with cervical length, measured using transvaginal ultrasound, < 15 mm or within the 15–30 mm range along with a positive PartoSure test (Parsagen Diagnostics Inc., Boston, MA)^34,35^.

PPROM was diagnosed by visualization of pooled amniotic fluid in the posterior fornix of the vagina during examination in a sterile speculum. In cases of clinical uncertainty, PPROM was confirmed by the presence of insulin-like growth factor-binding proteins (Actim PROM test; Medix Biochemica, Kauniainen, Finland) in the vaginal fluid^35^.

Sample collection for this study was approved by the Institutional Review Board of the University Hospital Hradec (PPROM: June 2014, No. 201508 S07P; PTL: June 2015, No. 201408 I96L). All pregnant women provided written informed consent prior to the collection of body fluids. This retrospective nested study, as a part of the project “PERSONMED—the center for the development of personalized medicine in age-dependent diseases, preterm delivery, and the influence of exogenous and endogenous factors,” was approved by the Institutional Review Board of the University Hospital Hradec (February 2019, approval number 201902 S16P). All the included participants were white pregnant women, and all experiments were performed in accordance with the relevant guidelines and regulations. All procedures performed in this study were in accordance with the ethical standards of the Declaration of Helsinki.

### Amniotic and cervical fluid sampling

Paired fluid samples (amniotic fluid first, followed by cervical fluid) were collected at the time of admission from all pregnant women included in this study prior to the administration of antibiotics, tocolytics, and/or corticosteroids. The sampling of both body fluids has been previously described in detail^11,12^. In brief, amniotic fluid was obtained via transabdominal amniocentesis and divided into aliquots for: (i) IL-6 measurement (electrochemiluminescence immunoassay); (ii) aerobic/anaerobic cultures; (iii) species-specific PCR for *Ureaplasma spp.*, *Mycoplasma hominis*, and *Chlamydia trachomatis*; and (iv) broad-range 16S rRNA PCR with Sanger sequencing. The remaining amniotic fluid was used for research purposes—the sample was centrifuged at 300 × g for 15 min, and both the supernatant and pellet were stored at − 80 °C until analysis. Cervical fluid was collected at admission using a Dacron swab placed in the cervical canal for 20 s. The swab was then placed in 1.5 mL phosphate-buffered saline, shaken for 20 min, centrifuged (300 × *g*, 15 min), and both supernatant and pellet were stored at − 80 °C until analysis.

### Clinical definitions

Intra-amniotic inflammation was defined as concentration of interleukin-6 in amniotic fluid ≥ 3000 pg/mL.  Microbial invasion of the amniotic cavity was defined as the presence of microorganisms and/or their nucleic acids in amniotic fluid. Intra-amniotic infection was defined as the concomitant presence of both intra-amniotic inflammation and microbial invasion of the amniotic cavity. Sterile intra-amniotic inflammation was defined as the presence of intra-amniotic inflammation without microbial invasion of the amniotic cavity. Colonization of the amniotic cavity was defined as the presence of microbial invasion of the amniotic cavity without intra-amniotic inflammation. Without intra-amniotic infection/inflammation was defined as the absence of both intra-amniotic inflammation and microbial invasion of the amniotic cavity.

### eMLST scheme

This study used aliquots of extracted *U.* spp. DNA from cervical and amniotic fluid samples of the pregnant women with PTL and cervical fluid samples of the women with PPROM, stored at − 80 °C until analysis. Amniotic fluid samples of *U.* spp. DNA from pregnant women with PPROM were not used in this study because the eMLST schemes were performed and their eST were identified in our previous study^18^.

Aliquots of *U.* spp. DNA, an eMLST scheme using primers targeting four housekeeping loci (*ftsH, rpl22, valS,* and *thrS)* and two putative virulence loci (*ureG* and *mba-np1*) was performed as outlined by Zhang et al.^16^. The public MLST (PubMLST) database was used to identify matches with existing alleles^36^. Novel alleles and eSTs were assigned with new alleles and eST numbers, respectively.

All phylogenetic analyses were performed using R Statistical Software v. 4.3.2^37^, employing functions within the *Biostrings*^38^, *Tidyverse*^39^, *ape*^40^, and *ggtree*^41^ packages. The concatenated sequences of the six specified genes for each eST retrieved from the PubMLST database were subjected to phylogenetic analyses. This analysis enabled the classification of new eSTs into clusters I (*U. parvum*) and II (*U. urealyticum*) and further categorization into subgroups according to previously published works (*U. parvum*: subgroups A, B, C, and D; *U. urealyticum*: subgroups 1 and 2)^16,17,25,26^.

### Statistical analyses

Demographic and clinical characteristics were compared using the nonparametric Mann–Whitney *U* or Kruskal–Wallis *H* tests, as appropriate, for continuous variables and were presented as median values (interquartile range (IQR]) Categorical variables were compared using Fisher’s exact or chi-square test, as appropriate, and were presented as numbers (%). The normality of the data was tested using the Anderson–Darling test. Exact *p-*values were used for comparisons between women with and without the concurrent presence of amniotic fluid *U.* spp. DNA because of the small sample size of the subset of pregnant women with concomitant amniotic fluid *U.* spp. DNA. The Bonferroni correction for multiple comparisons was used for the analysis of demographical and clinical characteristics. Differences were considered statistically significant at *p* < 0.05. All *p*-values were obtained using two-tailed tests, and all statistical analyses were performed using GraphPad Prism, version 8.1.1. for Mac OS X (GraphPad Software, San Diego, CA, USA).

## Supplementary Information

Below is the link to the electronic supplementary material.

Supplementary Material 1

Supplementary Material 2

Supplementary Material 3

Supplementary Material 4

Supplementary Material 5

Supplementary Material 6

## Data Availability

Demographic, clinical and metagenomic data that support the finding of this study have been deposited in the OSF repository (Center for open science). The data are available at https://osf.io (DOI\u000010.17605/OSF.IO/UVS74). Metagenomic data have been deposited in the ENA database ( https://www.ebi.ac.uk/ena/browser/view/PRJEB91153 ).
