# Supplementary material for: Characterization of cervical fluid Ureaplasma species in pregnant women with spontaneous preterm delivery
Source: Sci Rep. 2025 Aug 30;15:31997. doi: 10.1038/s41598-025-16612-2 (PMC12398508; doi:10.1038/s41598-025-16612-2)
Supplement: Supplementary file 1 — Supplementary Material 1 [file 41598_2025_16612_MOESM1_ESM.doc]

**Supplementary file - Table 4** Microbial features in the amniotic fluid of pregnant women with preterm labor with intact membranes and preterm prelabor rupture of membranes regarding the presence or absence of cervical fluid *Ureaplasma* spp. DNA.

| **Preterm labor with intact membranes** | |
| --- | --- |
| **The presence of cervical fluid *Ureaplasma* spp. DNA**  **(n=53)** | **The absence of cervical fluid *Ureaplasma* spp. DNA**  **(n=56)** |
| *Capnocytophaga ochracea, Fusobacterium nucleatum, Ureaplasma* spp. (n=1) | *Gardnerella vaginalis, Lactobacillus plantum* (n=1) |
| *Ureaplasma* spp. (n=7) | *Klebsiella pneumoniae, Streptococcus anginosus* (n=1) |
| *Burkholderia cepacia* (n=1) | *Fusobacterium nucleatum* (n=1) |
| *Lachnococcus lactis* (n=1) | *Heamophilus influenzae* (n=1) |
| *Lachnoanaerobaculum saburreum* (n=1) |  |
| Non-identifiable bacteria by sequencing (n=1) |  |
|  | |
| **Preterm prelabor rupture of membranes** | |
| **The presence of cervical fluid *Ureaplasma* spp. DNA**  **(n=38)** | **The absence of cervical fluid *Ureaplasma* spp. DNA**  **(n=31)** |
| *Corynebacterium tuberculostearicum, Dermabacter hominis, Staphylococcus epidermidis* (n=1) | *Anaerococcus tetradius* (n=1) |
| *Gardnerella vaginalis, Sneathia sanquinegens* (n=1) | *Haemophilus influenzae* (n=1) |
| *Gardnerella vaginalis, Ureaplasma* spp. (n=1) | *Lactobacillus iners* (n=1) |
| *Streptococcus hominis, Ureaplasma* spp. (n=1) | *Lactobacillus jenseni* (n=1) |
| *Ureaplasma* spp. (n=18) | *Streptococcus agalactiae* (n=1) |
| *Haemophilus influenzae* (n=2) | *Streptococcus angionosus* (n=1) |
| *Chlamydia trachomatis* (n=1) |  |
| *Peptostreptococcus stomatis* (n=1) |  |
