# Supplementary material for: Characterization of cervical fluid Ureaplasma species in pregnant women with spontaneous preterm delivery
Source: Sci Rep. 2025 Aug 30;15:31997. doi: 10.1038/s41598-025-16612-2 (PMC12398508; doi:10.1038/s41598-025-16612-2)
Supplement: Supplementary file 3 — Supplementary Material 3 [file 41598_2025_16612_MOESM3_ESM.doc]

**Supplementary file - Table 1** Demographical and clinical characteristics of the pregnant women with preterm labor and intact membranes regarding the presence and absence of cervical fluid *Ureaplasma* spp. DNA.

| Characteristic | The presence of *Ureaplasma* spp.  (n=53) | The absence of *Ureaplasma* spp.  (n=56) | *P-*value |
| --- | --- | --- | --- |
| Maternal age [years, median (IQR)] | 28 (24-31) | 28 (26-30) | 0.43 |
| Nulliparous [number (%)] | 33 (62%) | 32 (57%) | 0.70 |
| Smoking [number (%)] | 12 (23%) | 4 (7%) | **0.03** |
| Pre-pregnancy body mass index [kg/m^2^, median (IQR)] | 24.8 (22.4-24.9) | 24.7 (22.1-28.7) | 0.87 |
| Gestational age at sampling [weeks + days, median (IQR)] | 29+2 (26+0-32+0) | 31+1 (27+0-33+2) | 0.19 |
| Gestational age at delivery [weeks + days, median (IQR)] | 32+0 (28+0-35+3) | 33+6 (30+5-37+2) | 0.08 |
| Interval between amniocentesis and delivery [days, median (IQR)] | 4 (1-31) | 11 (0-37) | 0.52 |
| Intra-amniotic infection [number (%)] | 12 (23%) | 4 (7%) | **0.03** |
| Sterile intra-amniotic inflammation [number (%)] | 18 (34%) | 7 (13%) | **0.01** |
| Without intra-amniotic infection/inflammation [number (%)] | 23 (43%) | 45 (80%) | **<0.0001** |
| *Ureaplasma* spp. in amniotic fluid [number (%)] | 8 (15%) | 0 (0%) | **0.002** |
| CRP levels at admission [mg/L, median (IQR)] | 7.1 (3.7-18.0) | 5.2 (3.0-11.4) | 0.16 |
| WBC count at admission [x10^9^ L, median (IQR)] | 13.8 (10.8-16.9) | 12.2 (9.4-16.5) | 0.28 |
| Administration of corticosteroids [number (%)] | 46 (87%) | 45 (80%) | 0.44 |
| Administration of antibiotics [number (%)] | 34 (64%) | 26 (46%) | 0.08 |
| Spontaneous vaginal delivery [number (%)] | 42 (79%) | 45 (80%) | 1.00 |
| Cesarean section [number (%)] | 11 (21%) | 10 (18%) | 0.81 |
| Forceps/vacuumextraction delivery [number (%)] | 0 (0%) | 1 (2%) | 1.00 |
| Birth weight [grams, median (IQR)] | 1,840 (990-2,365) | 2,065 (1,648-2,878) | 0.08 |
| Apgar score <7; 5 minutes [number (%)] | 5 (9%) | 7 (13%) | 0.76 |
| Apgar score <7; 10 minutes [number (%)] | 4 (8%) | 5 (9%) | 1.00 |

Abbreviations:

CRP, C-reactive protein

IQR, interquartile range

WBC, white blood cells

Continuous variables, presented as median (interquartile range), were compared using a nonparametric Mann-Whitney *U* test. Categorical variables, presented as number (%), were compared using Fisher’s exact test. Statistically significant results are marked in bold.
