# Supplementary material for: Characterization of cervical fluid Ureaplasma species in pregnant women with spontaneous preterm delivery
Source: Sci Rep. 2025 Aug 30;15:31997. doi: 10.1038/s41598-025-16612-2 (PMC12398508; doi:10.1038/s41598-025-16612-2)
Supplement: Supplementary file 4 — Supplementary Material 4 [file 41598_2025_16612_MOESM4_ESM.doc]

**Supplementary file - Table 2** Demographical and clinical characteristics of the pregnant women with preterm prelabor rupture of membranes regarding the presence and absence of cervical fluid *Ureaplasma* spp. DNA

| Characteristic | The presence of *Ureaplasma* spp. DNA  (n=38) | The absence of *Ureaplasma* spp. DNA  (n=31) | *P-*value |
| --- | --- | --- | --- |
| Maternal age [years, median (IQR)] | 30 (26-34) | 31 (28-35) | 0.21 |
| Nulliparous [number (%)] | 19 (50%) | 16 (52%) | 1.00 |
| Smoking [number (%)] | 8 (21%) | 0 (0%) | **0.007** |
| Pre-pregnancy body mass index [kg/m^2^, median (IQR)] | 25.1 (19.9-27.4) | 24.1 (21.2-27.2) | 0.73 |
| Gestational age at sampling [weeks + days, median (IQR)] | 32+4 (31+4-33+0) | 31+1 (27+5-32+6) | 0.06 |
| Gestational age at delivery [weeks + days, median (IQR)] | 32+5 (31+5-33+2) | 32+4 (30+5-33+4) | 0.99 |
| Latency from PPROM to amniocentesis [hours, median (IQR)] | 4 (2-7) | 5 (2-8) | 0.49 |
| Latency from PPROM to delivery [hours, median (IQR)] | 45 (14-86) | 166 (35-334) | **0.003** |
| Intra-amniotic infection [number (%)] | 14 (37%) | 4 (13%) | **0.03** |
| Sterile intra-amniotic inflammation [number (%)] | 1 (3%) | 2 (7%) | 0.58 |
| Colonization of the amniotic cavity [number (%)] | 12 (31%) | 2 (7%) | **0.01** |
| Without inflammation/microorganisms [number (%)] | 11 (29%) | 23 (74%) | **0.0003** |
| *Ureaplasma* spp. in amniotic fluid [number (%)] | 20 (53%) | 0 (0%) | **<0.0001** |
| CRP levels at admission [mg/L, median (IQR)] | 7.6 (3.3-18.9) | 5.0 (3.3-9.2) | 0.20 |
| WBC count at admission [x10^9^ L, median (IQR)] | 12.4 (10.2-15.0) | 11.6 (9.0-15.2) | 0.59 |
| Administration of corticosteroids [number (%)] | 37 (71%) | 30 (97%) | 1.00 |
| Administration of antibiotics [number (%)] | 38 (97%) | 31 (100%) | 1.00 |
| Spontaneous vaginal delivery [number (%)] | 28 (74%) | 16 (52%) | 0.08 |
| Cesarean section [number (%)] | 9 (23%) | 15 (48%) | **0.04** |
| Forceps/vacuumextraction delivery [number (%)] | 1 (3%) | 0 (0%) | 1.00 |
| Birth weight [grams, median (IQR)] | 1,880 (1,630-2,020) | 1,850 (1,420-2,140) | 0.95 |
| Apgar score <7; 5 minutes [number (%)] | 1 (3%) | 3 (10%) | 0.32 |
| Apgar score <7; 10 minutes [number (%)] | 1 (3%) | 2 (6%) | 0.58 |

Abbreviations:

CRP, C-reactive protein

IQR, interquartile range

PPROM, preterm prelabor rupture of membranes

WBC, white blood cells

Continuous variables, presented as median (interquartile range), were compared using a nonparametric Mann-Whitney *U* test. Categorical variables, presented as number (%), were compared using Fisher’s exact test. Statistically significant results are marked in bold.
