# Supplementary material for: Characterization of cervical fluid Ureaplasma species in pregnant women with spontaneous preterm delivery
Source: Sci Rep. 2025 Aug 30;15:31997. doi: 10.1038/s41598-025-16612-2 (PMC12398508; doi:10.1038/s41598-025-16612-2)
Supplement: Supplementary file 5 — Supplementary Material 5 [file 41598_2025_16612_MOESM5_ESM.doc]

**Supplementary file - Table 5. Expanded sequence types of *Ureaplasma* spp. DNA isolated from paired cervical and amniotic fluids from pregnant women with preterm labor with intact membranes divided into the subgroups of *Ureaplasma* spp. DNA.**

| **UPA A** | | | **UPA B** | | | **UPA C** | | | **UPA D** | | | **UUR 1** | | | **UUR 2** | | |
| --- | --- | --- | --- | --- | --- | --- | --- | --- | --- | --- | --- | --- | --- | --- | --- | --- | --- |
| **eST** | **Fluid** | | **eST** | **Fluid** | | **eST** | **Fluid** | | **eST** | **Fluid** | | **eST** | **Fluid** | | **eST** | **Fluid** | |
|  | **cervical**  **n (%)** | **amniotic**  **n (%)** |  | **cervical**  **n (%)** | **amniotic**  **n (%)** |  | **cervical**  **n (%)** | **amniotic**  **n (%)** |  | **cervical**  **n (%)** | **amniotic**  **n (%)** |  | **cervical**  **n (%)** | **amniotic**  **n (%)** |  | **cervical**  **n (%)** | **amniotic**  **n (%)** |
| 17 | 4 (21%) | 1 (100%) | 1 | 1 (20%) |  | 2 | 1 (4%) |  | 172 | 1 (100%) |  | **257** | 1 (33%) |  | **258** | 1 (100%) |  |
| 20 | 3 (16%) |  | 16 | 2 (40%) | 1 (100%) | 4 | 3 (13%) |  |  |  |  | **259** | 1 (33%) |  |  |  |  |
| 41 | 5 (26%) |  | 239 | 2 (40%) |  | 192 | 2 (8%) |  |  |  |  | **261** | 1 (33%) |  |  |  |  |
| **49** | 1 (5%) |  |  |  |  | 217 | 2 (8%) | 1 (33%) |  |  |  |  |  |  |  |  |  |
| 250 | 5 (26%) |  |  |  |  | 234 | 2 (8%) | 1 (33%) |  |  |  |  |  |  |  |  |  |
| **260** | 1 (5%) |  |  |  |  | 244 | 5 (21%) | 1 (33%) |  |  |  |  |  |  |  |  |  |
|  |  |  |  |  |  | **245** | 2 (8%) |  |  |  |  |  |  |  |  |  |  |
|  |  |  |  |  |  | **246** | 1 (4%) |  |  |  |  |  |  |  |  |  |  |
|  |  |  |  |  |  | **247** | 1 (4%) |  |  |  |  |  |  |  |  |  |  |
|  |  |  |  |  |  | **252** | 1 (4%) |  |  |  |  |  |  |  |  |  |  |
|  |  |  |  |  |  | 253 | 1 (4%) |  |  |  |  |  |  |  |  |  |  |
|  |  |  |  |  |  | **254** | 1 (4%) |  |  |  |  |  |  |  |  |  |  |
|  |  |  |  |  |  | **255** | 1 (4%) |  |  |  |  |  |  |  |  |  |  |
|  |  |  |  |  |  | **256** | 1 (4%) |  |  |  |  |  |  |  |  |  |  |

Abbreviations:

eST, expanded sequence type

UPA A, *U. parvum* subgroup A

UPA B, *U. parvum* subgroup B

UPA C, *U. parvum* subgroup C

UPA D, *U. parvum* subgroup D

UUR 1, *U. urealyticum* subgroup 1

UUR 2, *U. urealyticum* subgroup 2

Novel eST identified in this study is in bold.
