# Supplementary material for: Characterization of cervical fluid Ureaplasma species in pregnant women with spontaneous preterm delivery
Source: Sci Rep. 2025 Aug 30;15:31997. doi: 10.1038/s41598-025-16612-2 (PMC12398508; doi:10.1038/s41598-025-16612-2)
Supplement: Supplementary file 6 — Supplementary Material 6 [file 41598_2025_16612_MOESM6_ESM.doc]

**Supplementary file - Table 6** Expanded sequence types of *Ureaplasma* spp. DNA isolated from paired cervical and amniotic fluids from pregnant women with preterm prelabor rupture of membranes divided based on the subgroups of *Ureaplasma* spp. DNA.

| **UPA A** | | | **UPA B** | | | **UPA C** | | | **UUR 1** | | |
| --- | --- | --- | --- | --- | --- | --- | --- | --- | --- | --- | --- |
| **eST** | **Fluid** | | **eST** | **Fluid** | | **eST** | **Fluid** | | **eST** | **Fluid** | |
|  | **cervical**  **n (%)** | **amniotic**  **n (%)** |  | **cervical**  **n (%)** | **amniotic**  **n (%)** |  | **cervical**  **n (%)** | **amniotic**  **n (%)** |  | **cervical**  **n (%)** | **amniotic**  **n (%)** |
| 15 | 1 (8%) | 1 (14%) | 1 | 1 (17%) |  | 2 | 1 (10%) |  | 82 | 1 (17%) | 1 (25%) |
| 17 | 2 (17%) |  | 16 | 3 (50%) | 1 (33%) | 4 | 1 (10%) |  | 147 | 1 (17%) | 1 (25%) |
| 20 | 4 (33%) | 2 (29%) | 239 | 1 (17%) | 1 (33%) | 111 | 1 (10%) |  | 167 | 2 (33%) | 1 (25%) |
| 41 | 4 (33%) | 4 (57%) | 240 | 1 (17%) | 1 (33%) | 233 | 1 (10%) | 1 (20%) | 249 | 2 (33%) | 1 (25%) |
| 250 | 1 (8%) |  |  |  |  | 244 | 1 (10%) | 1 (20%) |  |  |  |
|  |  |  |  |  |  | 248 | 1 (10%) | 1 (20%) |  |  |  |
|  |  |  |  |  |  | 253 | 2 (20%) | 1 (20%) |  |  |  |
|  |  |  |  |  |  | 255 | 1 (10%) |  |  |  |  |
|  |  |  |  |  |  | 262 | 1 (10%) | 1 (20%) |  |  |  |

Abbreviations:

eST, expanded sequence type

UPA A, *U. parvum* subgroup A

UPA B, *U. parvum* subgroup B

UPA C, *U. parvum* subgroup C

UUR 1, *U. urealyticum* subgroup 1

.
